# Editorial of Special Issue “Surface-Functionalized Nanoparticles as Drug Carriers”

**DOI:** 10.3390/ijms20246352

**Published:** 2019-12-17

**Authors:** Buddhadev Layek, Jagdish Singh

**Affiliations:** 1 Department of Pharmaceutics, College of Pharmacy, University of Minnesota, Minneapolis, MN 55455, USA; 2 Department of Pharmaceutical Sciences, College of Health Professions, North Dakota State University, Fargo, ND 58108-6050, USA

**Keywords:** drug delivery, nanoparticles, surface-functionalized nanoparticles, bioavailability, controlled release

## Abstract

Safe and effective delivery of therapeutics at the target site is the key to successful therapy. Nanocarriers can offer significant advantages over conventional dosage forms. Over the decades, nanoparticles have been extensively used to increase bioavailability, improve solubility and stability, reduce toxicities, and facilitate the controlled release of therapeutics. Further, nanoparticles have often been surface-functionalized with a variety of ligands to enhance circulation half-life and increase target-specificity. Although nanotechnology has shown significant therapeutic benefits for multiple biomedical applications, limited nanoparticle-based formulations have progressed to clinical trials, and only a few have reached the pharmaceutical market. This editorial is an introduction to the special issue entitled Surface-Functionalized Nanoparticles as Drug Carriers. We outline the scope of the special issue, summarize the results and conclusions of the nine articles published in this issue, and provide perspective on the application of surface-functionalized nanoparticles in the drug delivery field.

Nanoparticles are emerging as powerful tools in biomedical applications, particularly in the field of drug delivery and biomedical imaging. With their unique physicochemical properties such as nanoscale size, large surface area to volume ratio, and high reactivity, nanoparticles can modulate the basic properties and bioactivity of drug payloads [1]. Over the decades, nanocarriers have been extensively investigated to increase bioavailability, improve stability, reduce toxicities, facilitate the controlled release, and site-specific delivery of therapeutics [2,3,4]. However, the efficacy of nanocarrier-based drug delivery systems is mostly dependent on their controlled interactions with biomolecules. Therefore, nanoparticles have often been surface-functionalized with a variety of ligands, not only to impart site-specificity and increase cell penetration but also to provide stealth properties [5] and improve payload capacity [6]. For instance, the surface functionalization of nanoparticles has made remarkable advances in tumor-targeted delivery [7,8] and drug delivery across the blood-brain barrier [9,10]. Overall, nine articles published in this special issue highlight the recent developments in the fields of nanoparticle-based drug delivery systems.

Gonzalez-Garcinuno et al. [11] prepared levan-capped silver nanoparticles using an environmentally friendly technique and characterized for their antibacterial effect. These nanoparticles have shown potential bactericidal activity against both Gram-negative and Gram-positive bacteria. However, Gram-positive organisms were slightly less sensitive due to the presence of a thick peptidoglycan layer, which prevents the nanoparticle penetration inside the bacterial cytoplasm. The bacterial survival followed a single-hit/multiple-target model with a lethal silver dose of 6.8 ppm for *Escherichia coli* (Gram-negative) and 12.6 ppm for *Bacillus subtilis* (Gram-positive). The levan-capped nanoparticles were further incorporated into an alginate hydrogel that can be useful for diverse applications. 

Nanoparticles have been shown to improve the oral bioavailability of drugs while minimizing their toxicity to the gastrointestinal mucosa. However, the mechanism of nanoparticle transport across the intestinal mucosa is not fully understood. Thus, Ishii et al. [12] investigated the pathway for the transintestinal penetration of indomethacin-loaded solid nanoparticles (IND-NPs). Initially, IND-NPs were prepared by a bead mill method and evaluated their storage stability. Nanoparticles were capable of maintaining their nano-size order, and no degradation or decrease in nanoparticle numbers was observed after 30 days of storage in the dark at 20 °C. Further, no detectable precipitation or aggregation was observed in the same time period. The transport mechanism of IND-NPs was evaluated using Caco-2 cell monolayers and excised rat intestine in the presence of different endocytosis inhibitors. Caco-2 cell monolayers and excised rat intestine were pretreated with 54 µM nystatin (caveolae-dependent endocytosis inhibitor), 40 µM dynasore (clathrin-dependent endocytosis inhibitor), 2 µM rottlerin (macropinocytosis inhibitor), or 10 µM cytochalasin D (phagocytosis inhibitor) to inhibit specific endocytosis pathway and thermoregulated at 4 °C to arrest all energy-dependent endocytosis. Transintestinal penetration of IND-NPs was significantly reduced at 4 °C temperature. Studies with different pharmacological inhibitors demonstrated that only dynasore attenuated IND-NPs penetration in the jejunum, while in the ileum, both nystatin and dynasore inhibited nanoparticle penetration. Thus, the authors have concluded that nanoparticles were primarily taken up into the intestinal epithelium via energy-dependent endocytosis (clathrin-dependent endocytosis in the jejunum, and both caveolae-dependent and clathrin-dependent endocytosis in the ileum) and dissolved or diffused in the intestinal mucosa.

In their article, Faizan et al. [13] evaluated pharmaceutical clay montmorillonite (MMT) as carbon monoxide (CO)-releasing material (CORMat). The CO-releasing molecule-2 (CORM-2) [Ru (CO)_3_Cl_2_]_2_ was intercalated into the clay layers to minimize the toxicity of the organometallic segment. SEM and TEM images assured that MMT maintained its layered structure even after loading with the ruthenium compound. The energy-dispersive X-ray spectroscopy analysis demonstrated that the concentration of the ruthenium element in MMT was about 5%. The horse myoglobin assay confirmed the slow release of CO in the biological system. Furthermore, the excellent biocompatibility of CORMat showed its potential to be used as a CO delivery system for the treatment of Ulcerative Colitis.

Chemotherapy is one of the most effective and widely used treatment modalities in the majority of cancer types. However, conventional chemotherapeutics lack sufficient cancer selectivity and can damage both healthy and cancerous cells, causing severe side effects. Inorganic nano-drug delivery platforms (NDDPs) have been emerged as potential candidates in cancer therapy, alleviating the limitations of conventional chemotherapy. The review article by Naz et al. [14] delves into summarizing the recent advances in NDDPs for tumor-targeted delivery of chemotherapeutic agents. This article discusses the in-depth therapeutic potential of several promising inorganic NDDPs such as mesoporous silica nanoparticles, carbon nanotubes, superparamagnetic iron oxide nanoparticles, layered double hydroxides, and calcium phosphate nanoparticles in treating the various types of cancer. Anticancer drugs loaded inorganic NDDPs offer several advantages, including enhanced drug stability, prolonged systemic circulation, preferential accumulation in tumor tissue due to enhanced permeability and retention (EPR) effect, and sustained drug release in tumor cells. Thus, NDDPs can enhance the clinical outcomes of cancer therapy by improving drug efficacy and reducing the required dosage and toxicity. This review also highlights the emergent tumor-targeted inorganic NDDPs that could potentially be translated into clinical trials in the future.

Jang et al. [15] have developed polymeric nanoparticles to improve lymphatic delivery and minimize the toxicity of methotrexate (MTX). The MTX-loaded poly(lactide-co-glycolide) (PLGA) nanoparticles were prepared by a double emulsion solvent evaporation method. The particle size of the nanoparticles was significantly influenced by the various formulation parameters, including PLGA concentration, PVA concentration, oil-water phase volume ratio, and amount of the drug. Particle size increased as the PLGA concentration, PVA concentration, or amount of the drug increased while particles with the smallest size were attained when the oil-water phase volume ratio was 1:3. The average hydrodynamic diameter and encapsulation efficiency of the optimized nanoparticles (0.5% w/v PLGA concentration, 1% w/v PVA concentration, 3 mg MTX, and oil-water phase volume ratio of 1:3) were 163.7 ± 10.3 nm and 93.3 ± 0.5%, respectively. Nanoparticulate formulations showed sustained release profiles of MTX at various pH conditions and effectively inhibited cancer cell proliferation. Furthermore, MTX-loaded nanoparticles showed approximately two times higher area under the curve (AUC), longer half-life (t_1/2_), and two times lower plasma clearance (CL) than free MTX.

Surface-functionalized nanoparticles have been extensively utilized for site-specific delivery of active drug molecules. However, one of the critical challenges in the development of surface-modified nanoparticles is the accurate assessment of the number of ligands on the nanoparticle surface. Gauthier et al. [16] developed *N*-acetyl-D-galactosamine (GalNAc) functionalized nanostructured lipid carriers (NLC) for efficient targeting of asialoglycoprotein receptor (ASGPR) that are overexpressed on hepatocytes. Four different NLC formulations were manufactured using various molar percentages of GalNAc-functionalized surfactant (0%, 2%, 5%, and 14%). The amount of GalNAc present on the NLC surface was determined based on ultra-high-performance liquid chromatography separation and evaporative light-scattering detection method (UPLC-ELSD). This method allowed selective and precise quantification of GalNAc units present on the NLC surface. 

Dos Santos-Silva et al. [17] studied the effect of surface functionalization with sialic acid (SA) and cholesterol (Chol) on the cellular uptake and in vitro cytotoxicity of benznidazole-loaded cationic polymethylmethacrylate (PMMA) nanoparticles (BNZ NPs). An emulsification solvent evaporation method was used to prepare small (<200 nm) narrow-sized cationic nanoparticles with high drug-loading efficiency. Surface-functionalization with SA or Chol increased the particle size, reduced drug-loading efficiency, and slightly decreased zeta potential of BNZ NPs. All formulations were nontoxic to normal kidney cells (HEK 293) and improved BNZ efficacy, compared to the free drug. The surface-functionalization of BNZ-NPs enhanced the cytotoxic effect in HeLa and HT-29 cells, with slightly better efficiency with the Chol-functionalization. 

Minimizing the risk of fetal exposure to medication and reducing off-target toxicity in mothers are the significant challenges in developing an effective therapy for pregnancy complications. In their review, Zhang et al. [18] discuss the recent developments in surface-functionalized nanoparticle-based delivery systems for targeted drug delivery in pregnancy complications. Surface-functionalized nanoparticles have shown great potential to facilitate placenta- and uterus-specific drug delivery while preventing transplacental passage, thereby improving the safety and efficacy of the treatment. However, the development of effective nanomedicines for pregnancy complications requires a detailed understanding of human placental anatomy and transplacental nanoparticle transport mechanism. Therefore, the authors first describe the anatomical structures and composition of the human placenta as well as the existing mechanisms of nanoparticle transport across the placenta. Nanoparticles conjugated with an anti-oxytocin receptors (OTR) antibody have efficiently delivered drug molecules to the uterus [19]. Several other placentae- and uterus-enriched molecules such as epidermal growth factor receptor, placental chondroitin sulfate A-binding receptor, integrins, and uterine have been used as molecular targets for site-specific drug delivery in pregnancy complications. The authors, therefore, proposed that placenta-specific exosomes and surface-modified exosomes could be served as potential tools to treat pregnancy complications.

Finally, Shreffler et al. [20] comprehensively review the primary challenges for translating nanomedicines into the clinic. The nanoparticle–immune system interactions often result in the rapid clearance of the nanoparticles and impose an obstacle for the clinical translation of nanoparticle-based systems. Surface-functionalization such as PEGylation or acetylation, surface protein addition, overall surface charge, and pH of the formulation are known to play critical roles in delineating physiological target and enhance the retention time of nanoparticles within the body. However, the surface modification of the nanoparticles must be selected carefully to achieve the desired efficacy. Furthermore, the precise prediction of efficacy, retention, clearance, and safety profiles of nano-formulations in human patients by using appropriate preclinical animal models is equally important. Therefore, the authors have provided some general instruction in selecting both appropriate nanoparticle surface modification approaches and suitable preclinical animal models to evaluate the immune response to those modifications.

Although nanotechnology has shown significant therapeutic benefits for multiple biomedical applications, limited nanoparticle-based formulations have advanced from preclinical to clinical phases, and only a few are available in clinics. However, the development of cutting-edge manufacturing processes, better understanding of the fate of nanoparticles in a physiological environment, site-specific targeting, and use of appropriate in vitro and in vivo evaluation techniques could enhance the clinical translation rate of nanoformulations. Hence, we firmly believe this issue will enrich the knowledge and understanding of the researchers from both industry and academia on nanoparticle-based formulations used in drug delivery. 
